# Life satisfaction in patients with long-term non-malignant pain – relating LiSat-11 to the Multidimensional Pain Inventory (MPI)

**DOI:** 10.1186/1477-7525-6-70

**Published:** 2008-09-23

**Authors:** Annika J Silvemark, Håkan Källmén, Kamilla Portala, Carl Molander

**Affiliations:** 1 Department of Neuroscience, Rehabilitation Medicine, Uppsala University. Uppsala University Hospital, SE-751 85 Uppsala, Sweden; 2 Department of Psychology, Uppsala University, Sweden; 3 Department of Neuroscience, Psychiatry, University Hospital, Uppsala University, SE- 751 85 Uppsala, Sweden

## Abstract

**Background:**

The West-Haven Multidimensional Pain Inventory (MPI) can be used to describe behavioural and psychosocial consequences of long-term pain but little is known about how MPI items and MPI subgroups relate to goals that patients find important in rehabilitation. Life satisfaction measured by the LiSat-11 checklist can be defined as an individual's perception of the difference between his reality and his needs or wants. This difference can be considered a "goal achievement gap". This study investigates the relation of MPI to LiSat-11 with the aim to explore the possibility that LiSat-11 can be used to measure pain rehabilitation outcomes that are important from the patients' view.

**Methods:**

Participators were patients (n = 294) referred to the Pain and Rehabilitation Clinic in Uppsala, Sweden. Measures used were LiSat-11, MPI and its Swedish version MPI-S. LiSat-11 domains were correlated to MPI scales. Cluster analysis was used to demonstrate MPI-S subgroups. Analysis of variance followed by post-hoc analysis was used to investigate life satisfaction in the three MPI-S subgroups.

**Results:**

The strongest positive correlation were found for the LiSat-11 domains/MPI scales: psychological health/life control and contacts/social activities, and the strongest negative correlation for: psychological health/affective distress, partner relationship/punishing responses, somatic health/interference and leisure/interference. None or only little correlation was found between MPI scale pain severity and most LiSat-11 domains and satisfaction with life as a whole. Among the MPI-S subgroups, adaptive copers generally had better life satisfaction than the dysfunctional and the interpersonally distressed.

**Conclusion:**

Pain severity alone is a rather poor predictor of low life satisfaction. MPI and LiSat-11 partly supplement each other as tools to describe how functional impairments relate to life satisfaction domains, which may be relevant for identifying domains which the patients find important to improve. Furthermore, differences in life satisfaction between the MPI-S subgroups may help to identify functional domains that may be of particular importance in specialised rehabilitation programs.

## Background

The prevalence of long term non malignant pain, defined as VAS > 5/10, has recently been reported to be 18% in the Swedish population[1]. Recent studies have shown that multiprofessional rehabilitation programs can provide valuable help (see[2]), but there is little systematic knowledge of patient selection criteria to enter programs and how programs should be designed to meet the needs of the individual patient. Negative functional consequences of long-term pain do not necessarily require rehabilitation unless they are associated with subjective needs of the patient.

The concept of life satisfaction (LiSat) focus on the individual's perception of the difference between the subjective reality and needs or wants regarding several important domains of functioning and activity/participation. This difference can be considered a "goal achievement gap" [3-6]. The LiSat-11 checklist developed by Fugl-Meyer and Fugl-Meyer has been tested in a large reference group from the normal population[6] and is included in the Swedish National Quality Registry for Pain Rehabilitation (NRS) and therefore offers good opportunities for comparisons between subgroups of pain patients and treatments on a national level. We have found[7] that life satisfaction measured by LiSat-11 is considerably lower in patients with long-term pain than in a larger reference group from the general population.

In addition to low life satisfaction and physical impairments, long-term pain is in general linked to a number of psychosocial and behavioural consequences. These can be demonstrated by using a questionnaire such as the West Haven Yale Multidimensional Pain Inventory (MPI). This instrument has been shown to have good psychometric properties[8]. MPI is also included in NRS (see above). Using MPI or MPI-S, three reliable and valid subgroups were revealed which seem to react and cope differently to pain when compared to each other; interpersonally distressed (ID) patients, dysfunctional (DYS) patients, and adaptive copers (AC) [9-12]. The ID patients had high pain severity, interference and affective distress and scored low on social support and solicitous responses but high on punishing responses from significant others. The DYS patients had high pain severity, interference and affective distress, and a rather low life-control but scored high on social support, solicitous responses and distracting responses. The AC patients had low pain severity, interference, affective distress and were low on punishing responses, and had better life-control than the others.

MPI is used to describe the behavioural and psychosocial functioning of the patient but so far it appears to be poorly known to what extent MPI scores are important for the individual patient in a rehabilitation program. One way of describing the importance of MPI scores would be to relate them to scores of LiSat-11 domains. Furthermore, if LiSat-11 can be correlated to MPI, then it might be possible to use LiSat-11 and MPI together as outcome measures in pain rehabilitation.

The aim of the present study has been to explore the relation of behaviour/psychosocial functioning to life satisfaction. We first study the relation of individual LiSat-11 domains to individual MPI scales, and second LiSat-11 domains in patients belonging to the above mentioned MPI-S subgroups (ID, DYS, AC). We had the following hypotheses: impairments shown by MPI scales are associated with low values on related LiSat-11 domains, intense pain is strongly associated with low life satisfaction, AC patients have higher life satisfaction than ID and DYS patients, and finally that ID patients are comparatively less satisfied with family life and partner relationship.

## Methods

Participating subjects were 294 consecutive patients (collected from 2002–2005) diagnosed with long-term non-malignant pain (> 6 months) and fulfilling the inclusion criteria (see below). Demographic data for patients are shown in table 1. Patients were referred from regional general practitioners, company doctors and specialist clinics to the Pain and Rehabilitation Clinic, University Hospital, Uppsala, Sweden. This clinic is well established and has a long tradition in the evaluation and treatment of patients with long-term pain using multidisciplinary collaboration and approaches. The patients in this study are identical to those contributing to a companion study[7] in which life satisfaction in patients with long-term pain was compared to a Swedish reference group sampled from the normal population, and related to demographic data and pain severity.

**Table 1:** Demographic data

	Pain patients	
	
*Age*	Mean 38.1 SD 9.4	
*Gender*	No.	%
Females	193	66
Men	101	34
		
*Origin*		
Born in Scandinavia	239	81
Born outside Scandinavia	49	17
		
*Education*		
Compulsory school only	53	18
Upper secondary school	176	60
Higher education	51	17
		
*Source of income*^1^		
Salary	84	27
Sickness benefit	220	69
Sickness pension	13	4
Social allowance	3	1
*Pain severity estimated by MPI (0–6)*	Mean 4.2, SD 0.9, Median 4.3	
*Duration of pain*	Mean 2344 days, SD 2264	
*Pain localisation*^2^		
Neck	59	20
Shoulder and/or arm	38	15
Thoracic back	13	4
Lumbar back	24	8
Location varies	95	32
Other specified localisations	57	20

^1^Several sources of income are possible.

^2^Several pain localisations are possible.

Inclusion criteria were: age 18–64 years, ability to communicate in Swedish and to fill in medical questionnaires, and considered by the rehabilitation specialist to be in need of a multi-professional rehabilitation team (nurse, physician, physiotherapist, occupational therapist, psychologist, social counsellor) for their medical investigation. Patients with either depression or ongoing substance abuse so severe that they were judged to be unable to participate in the medical investigation by the rehabilitation team were excluded (external dropouts). We do not know how many these were. Furthermore, among the excluded patients were ten patients who did not fill in the questionnaires at all. The remaining 294 subjects filled in personal demographic data, and a life satisfaction checklist (LiSat-11) and Multidimensional Pain Inventory (MPI), see below. The frequencies of internal dropouts (did not answer all questions) were 9–25% for LiSat-11, and 8–18% for MPI. The final number of subjects that contributed full data to the analyses in the study was at least 75% for LiSat-11 and 82% for MPI.

The Life Satisfaction checklist (LiSat-11)[6,13] consists of patients estimations of satisfaction with life as a whole as well as satisfaction in ten specific domains: vocation, economy, leisure, contacts, sexual life, activities of daily living (ADL), family life, partner relationship, somatic health, psychological health. The construct validity of LiSat-11 has been shown to be acceptable by using a principal components analysis forming 4 components, whereof 3; "Closeness" (Chronbach's α = 0.79), "Health" (Chronbach's α = 0.66) and "Spare time" (Chronbach's α = 0.68), had acceptable internal consistency. One subscale; "Provision" did not show an acceptable consistency (Chronbach's α = 0.57)[6,13]. The responses were made on a 6 point Likert-scale: 1 = very dissatisfied; 2 = dissatisfied, 3 = rather dissatisfied, 4 = rather satisfied, 5 = satisfied, 6 = very satisfied.

The MPI is a self-report questionnaire on psychological, social and behavioural aspects of chronic pain, divided in 3 sections ("impact of pain on patients life", "responses of others to patients communication of pain", and "participation in common daily activities"; in all 61 items distributed on 13 scales). The English original version was shown to have strong psychometric properties[8]. The 13 scales are: pain severity, interference, life control, affective distress, support, punishing responses, solicitous responses, distracting responses, household chores, outdoor work, activities away from home, social activities, and general activities. The responses are given on a 7 point numeric scale. A Swedish translation of the original English version provided by the NRS (see above) committee, including all 61 questions, was used in the first part of the present study to relate LiSat-11 to individual MPI scales. However, Bergström and co-workers[14,15] showed that for their modified Swedish version of MPI, the MPI-S, only the 2 first sections (impact and responses, see above) showed an acceptable factor structure, whereas the scales in the third section (activities) did not. It was suggested, therefore, that this part is used only for assessing the general activity level. In addition, some items in the first two sections showing weak reliability were also deleted in the MPI-S. For this reason Bergström and co-workers used the shorter MPI-S in their cluster analysis, confirming the previously mentioned subgroups: AC, ID, DYS. In our analysis of those subgroups in relation to life satisfaction, we therefore used the MPI-S (second part of this study).

Data analyses were made by using SPSS 11.5 software. As life satisfaction followed an approximate normal distribution we used parametric statistics in the calculations. Unpaired T-test was used to test the hypothesis of equal life satisfaction among those who estimated an average pain above median, and those who scored below median on pain severity (part of the MPI). The statistical significance level was set to 0.05. Correction of the significance level when having multiple tests was made by using Bonferroni's method.

Internal reliability of MPI and LiSat-11 were calculated by using Cronbach's alpha. Pearson's product-moment correlations were calculated to evaluate the covariance between domain specific life-satisfaction and MPI items (all three sections), including estimation of how pain severity affects different aspects of life satisfaction.

Scores from the 34 items MPI-S were z-transformed to reach a standard with mean = 0 and standard deviation = 1. A non-hierarchical clustering procedure (K-means cluster analysis, SPSS package 11.5) was performed on the z-transformed scores using all patients in the sample. Since it has been shown that a solution of three clusters of MPI-S items was appropriate among pain patients[9,10], this number of clusters were extracted in the analysis. The hypothesis of equal centroids from the 8 MPI-S scales (34 items from MPI sections 1 and 2, but excluding sections 3), referring to the MPI subgroups (ID, DYS, AC), was tested by using Multivariate Analysis of Variance (MANOVA). The hypothesis of differences between the three subgroups was tested using univariate ANOVA. Pair-wise comparisons between subgroups were made using Scheffé's method.

A Swedish ethical committee has previously confirmed that the national use of the questionnaires in the NRS-register is in accordance with applicable legislation, and the local ethical committee found that the design of the present study did not require further formal ethical consideration (Dnr 2004: M-381).

## Results

### Internal reliability

The internal consistency of the LiSat-11 checklist was good (Cronbach's α = 0.82). The internal consistency of the MPI-Scales in this study were good in section 1 (impact; Chronbach's alpha's 0.70–0.87) and in section 2 (responses of others; Cronbach's alpha's 0.75 – 0.85), but lower in section 3 (activity; Cronbach's alpha's 0.50 – 0.82). The subscales "activity away from home" (alpha = 0.50) and "social activities" (alpha= 0.57) did not show an acceptable internal reliability.

### Relation of individual LiSat-11 domains to individual MPI scales

Pearson product-moment correlations between LiSat-11 domains and MPI scales (all three sections) are shown in table 2. Most correlations were rather weak. High positive correlations were noted for the following LiSat-11/MPI scale pairs: psychological health/life control, and contacts/social activities, and negative correlations for psychological health/affective distress, partner relationship/punishing responses, somatic health/interference and leisure/interference.

**Table 2:** Correlations between LiSat-11 domains and MPI scales.

	*LiSat-11 domains*										
*MPI scales*	1	2	3	4	5	6	7	8	9	10	11
*Section 1*											
Pain Severity	-0.15	-0.15	-0.15	-0.18	-0.15	-0.05	-0.19	-0.07	-0.03	**-0.32**	-0.20
Interference	**-0.36**	-0.28	-0.25	**-0.46**	**-0.40**	**-0.30**	**-0.30**	-0.20	-0.18	**-0.47**	**-0.34**
Life control	**0.33**	0.09	0.24	**0.30**	**0.35**	0.20	0.24	0.27	0.26	**0.35**	**0.50**
Affective distress	**-0.34**	-0.14	-0.28	**-0.35**	**-0.39**	-0.25	-0.12	**-0.35**	**-0.34**	-0.28	**-0.59**
Social support	0.10	0.05	0.15	-0.01	-0.06	0.18	-0.04	**0.34**	**0.36**	-0.09	0.06
*Section 2*											
Punishing Responses	-0.27	-0.12	-0.20	-0.14	-0.26	-0.25	0.02	-0.28	**-0.50**	0.05	-0.21
Solicitous Responses	0.19	0.10	0.12	0.01	0.02	0.16	-0.17	0.29	**0.32**	-0.02	0.09
Distracting Responses	0.12	0.01	0.06	-0.05	-0.02	0.17	-0.10	0.20	0.24	0.03	0.10
*Section 3*											
Household Chores	0.13	0.09	0.01	0.23	0.15	0.12	0.23	0.19	0.19	0.02	0.19
Outdoor work	0.06	0.02	-0.04	0.14	0.21	0.08	0.15	0.05	0.08	0.14	0.09
Activities away from home	**0.31**	0.11	0.17	**0.32**	**0.34**	0.25	0.11	**0.34**	0.26	0.14	0.27
Social activities	**0.34**	0.03	0.08	**0.32**	**0.46**	0.22	0.08	0.29	**0.31**	0.06	0.28
General Activity Level	0.28	0.09	0.07	**0.35**	**0.40**	0.22	0.22	**0.30**	0.29	0.12	0.29

In bold face, domains explaining at least 9% (r_xy_^2^) of the variance. Pearson product moment correlation r_xy_.

MPI = Multidimensional Pain Inventory. LiSat-11 = Life satisfaction checklist. LiSat-11 domains: 1, satisfaction with life as a whole; 2, vocation; 3, economy; 4, leisure; 5, contacts; 6, sexual life; 7, activities of daily living (ADL); 8, family life; 9, partner relationship; 10, somatic health; 11 psychological health.

Patients who scored pain severity below the median value (4.3/6 = max value) on the MPI scale also scored higher on the following LiSat-11 domains compared to those who scored above the median value: leisure (t = 3.17 df = 261, P = 0.002), contacts (t = 2.46 df = 262, P = 0.015), sexual life (t = 2.50 df = 262, P = 0.013), somatic health (t = 4.27 df = 261, p < 0.001) and psychological health, (t = 3.65 df = 260, p < 0.001). There was no statistically significant association, however, between pain severity and satisfaction with life as whole. After decreasing the level of significance due to multiple comparisons by using the method of Bonferroni, satisfaction in the domains somatic health and psychological health were still significantly better among the patients who scored pain severity below the median value.

### Life satisfaction in MPI-S subgroups

The cluster analysis divided 272 of the 294 patients into the 3 subgroups. The remaining 22 patients could not be placed in a cluster, mainly due to missing data. The hypothesis of equal centroids from the 8 MPI-S scales (34 items from MPI sections 1 and 2, but excluding section 3), referring to the MPI subgroups (ID, DYS, AC), was tested by Multivariate Analysis of Variance (MANOVA). Wilks' lambda was 0.014 (p < 0.001), showing significant differences between the scale centroids. Follow-up univariate F-tests of the 8 MPI-S scales showed that significant differences existed between the 3 subgroups on all scales (table 3).

**Table 3:** Means and standard deviations of the 8 MPI-S scales in each MPI subgroup.

MPI-S scale	Cluster	Mean	SD	F	DF	p <
Pain severity	ID	4.34	0.79	21.84	2/245	0.001
	DYS	4.44	0.85			
	AC	3.37	0.85			
Interference	ID	4.60	0.73	85.68	2/245	0.001
	DYS	4.49	0.72			
	AC	2.84	0.62			
Life control	ID	2.41	0.94	29.11	2/245	0.001
	DYS	2.63	1.00			
	AC	3.89	0.84			
Affective distress	ID	3.75	1.14	33.32	2/245	0.001
	DYS	3.45	1.07			
	AC	1.94	1.13			
Social support	ID	3.06	1.27	56.59	2/245	0.001
	DYS	4.61	0.96			
	AC	3.69	1.48			
Punishing responses	ID	2.79	1.36	57.09	2/245	0.001
	DYS	1.01	0.98			
	AC	1.25	1.34			
Solicitous response	ID	1.68	0.94	78.69	2/245	0.001
	DYS	3.59	1.13			
	AC	2.29	1.22			
Distract responses	ID	1.29	0.96	58.22	2/245	0.001
	DYS	2.91	1.07			
	AC	2.04	1.13			

Univariate ANOVA's and p-values. ID = interpersonal distressed, DYS = dysfunctional, AC = active copers.

Pair-wise comparisons between subgroups using Scheffé's method showed that the means corresponding to the AC patients (n = 40) were significantly lower on Pain Severity than both ID patients (n = 83; p < 0.001) and DYS patients (n = 149; p < 0.001). AC patients also scored significantly lower on Interference and Affective Distress than both ID and DYS patients (all p < 0.001) and higher on Life Control (both p < 0.001). This confirms the construct validity of AC patients.

The DYS patients and ID patients scored similarly on Pain Severity, Interference and Affective Distress, and scored significantly higher on these scales than the AC patients (all p < 0.001). The score for the DYS patients on Life Control was significantly lower than for AC patients p < 0.001) but similarly to ID patients. However, DYS patients scored significantly higher than the other subgroups on Social Support, Solicitous Responses and on Distracting Responses (all p < 0.02). The result supports the construct validity of DYS patients.

The ID patients scored similarly to DYS patients but significantly higher than AC patients on Pain Severity, Interference, and Affective Distress (all p < 0.001). They scored significantly lower on Life Control and on Social Support than AC patients (both p < 0.001). They also scored significantly lower on Solicitous and Distracting Responses than the other two subgroups (p < 0.030), and higher on Punishing Responses (both p < 0.001). This confirms the construct validity of ID patients.

Significant differences in life satisfaction were found when the three MPI subgroups were compared, both for Life as a whole and for each domain of LiSat-11 (one-way ANOVA; table 4 and 5). Paired post hoc comparisons using Sheffé's method showed that AC patients were significantly more satisfied than the ID and DYS patients with life as whole and in all LiSat-11 domains (all p < 0.03), except for the domains family life and partner relationship for which AC scored higher than ID, but not compared to DYS. Furthermore, significant differences were found between the ID and DYS patients in the LiSat-11 domains economy, sexual life, family life and partner relationship, but not in satisfaction with vocation, leisure, contacts, daily activities, somatic health and psychological health.

**Table 4:** Mean and standard deviation for LiSat in the 3 MPI-S subgroups ID, DYS, AC.

Satisfaction with	MPI-S Subgroups	Mean	SD	F	Df	P <
Life as whole	ID	3.14	1.26	18.47	2/261	0.001
	DYS	3.65	1.03			
	AC	4.45	1.15			
Vocation	ID	2.00	1.30	9.59	2/253	0.001
	DYS	2.39	1.49			
	AC	3.24	1.46			
Economy	ID	2.46	1.24	20.95	2/264	0.001
	DYS	3.21	1.38			
	AC	4.10	1.30			
Leisure	ID	2.83	1.25	15.30	2/262	0.001
	DYS	3.04	1.17			
	AC	4.08	1.23			
Contacts with friends and acquaintances	ID	3.60	1.31	7.48	2/264	0.001
	DYS	3.91	1.29			
	AC	4.55	1.06			
Sexual life	ID	2.87	1.38	13.16	2/249	0.001
	DYS	3.57	1.51			
	AC	4.38	1.52			
ADL	ID	4.09	1.21	22.00	2/264	0.001
	DYS	3.97	1.27			
	AC	5.35	0.77			
Family life	ID	4.01	1.15	16.78	2/222	0.001
	DYS	4.77	1.07			
	AC	5.27	1.05			
Partner relationship	ID	3.78	1.29	30.35	2/216	0.001
	DYS	5.03	1.04			
	AC	5.20	1.00			
Somatic health	ID	2.06	1.00	26.24	2/263	0.001
	DYS	2.22	1.11			
	AC	3.50	1.16			
Psychological health	ID	3.16	1.28	19.86	2/262	0.001
	DYS	3.57	1.21			
	AC	4.65	1.14			

One-way ANOVAs, F and df and p-values.

**Table 5:** Post-hoc comparisons of life satisfaction between the three MPI-S subgroups.

**Satisfaction with**	**MPI-S subgroups**	
Life as whole	AC>ID, DYS	DYS≈ID*
Vocation	AC>ID, DYS	DYS≈ID
Economy	AC>ID, DYS	DYS>ID
Leisure	AC>ID, DYS	DYS≈ID
Contacts with friends and acquaintances	AC>ID, DYS	DYS≈ID
Sexual life	AC>ID, DYS	DYS>ID
Daily activites	AC>ID, DYS	DYS≈ID
Family life	AC>ID	DYS>ID
Partner relationship	AC>ID	DYS>ID
Somatic health	AC>ID, DYS	DYS≈ID
Psychological health	AC>ID, DYS	DYS≈ID

*DYS>ID nearly significant. ≈ denotes subgroups for which statistically significant difference could not be established. Sheffés method, p < 0.05.

## Discussion

The results of the present study showed that the internal consistency of the LiSat-11 checklist was acceptable and that the internal consistency of the MPI-scales were acceptable in section 1 and 2 but not in section 3 (activities). The strongest positive correlations were found for LiSat-11 domain/MPI scale: psychological health/life control and contacts/social activities, and the strongest negative correlations for: psychological health/affective distress, partner relationship/punishing responses, somatic health/interference and leisure/interference. Patients reporting pain severity below the median level reported higher life satisfaction on LiSat-11 somatic health and psychological health, but not on satisfaction with life as a whole. The internal consistency was confirmed for all three MPI-S subgroups: AC, ID, DYS. Finally, patients belonging to the MPI-S subgroup "active coopers" had higher satisfaction than "interpersonally distressed" and "dysfunctional" on most LiSat-11 domains.

### Methodological considerations, strengths and limitations

More than 20% among the LiSat-11 dropouts did not respond to questions about family life and partner relationship. One possibility is that respondents who were single did not know how to respond to these questions. The other dropouts were comparatively fewer.

Regarding MPI, patients in this study responded inconsistently (low Chronbach's alpha's) on two of the scales, "activities away from home" (0.50) and "social activities" (0.57). For this reason, and in accordance with the conclusion of Bergström and collaborators[14] who used only the first two sections of the original MPI in their modified shorter Swedish version MPI-S (34 items), we think that it is possible to omit the third section. We did not do this in the first part of this study when we related LiSat-11 domains to individual MPI scales for two reasons: first we were interested in exploring the relation of LiSat-11 to individual MPI scales, and second we considered the possibility that removing of selected questions would bias the responses of the remaining questions as a greater problem, a phenomenon called "framing"[16,17]. However, in order to be able to compare our results with a previous Swedish study by Bergström and co-workers[10] we too used only the first two sections in the cluster analysis when the three MPI subgroups IP, AC, and DYS were created. Even though it can not be excluded that parts of the links that formed the subgroups was related to factors such as gender, age, extent somatic pathology, previous studies indicate that these do not seem to be important[9,10]. Due to the non-random sample the external validity of our results could be questioned.

### Health Related Quality of Life(HRQL) and Life Satisfaction, general aspects

There are several different questionnaires available to measure HRQL; generic instruments such as Short-form 36 (SF-36)[18], Euro-Qol (EQ-5D)[19], Nottingham Health Profile[20], and instruments designed specifically for patients with long-term pain such as Oswestry Disability Questionnaire[21], Western Ontario and McMaster Universities Osteoarthritis index[22,23].

Life satisfaction as measured by the LiSat-11 is separate from the medical observer-treatment tradition used in most quality of life instruments. It reflects the need/want perception of the patient. The "need/want" in LiSat-11 is a transformation from perceptions of dissatisfaction or suboptimal satisfaction with several important aspects of the human life situation, some of which are related to functioning. Helping the patient to become more satisfied with those domains may require not only medical, but also psychological and social interventions.

Both LiSat-11 and MPI are multidimensional constructs that refer to a person's perceived quality of her/his physical, psychological, social and existential functioning and can, in a broad sense, therefore be considered to be associated with of the HRQL family of instruments. However, whereas MPI measures the impact of pain on different aspects of the patients' life[8], the intent of LiSat-11 is to measure how satisfied the patient is with several important aspects of his/her life, including some functional aspects including relations to others. In that respect, MPI and LiSat-11 measure different dimensions and can therefore be claimed to supplement each other. MPI would characterise the pain- inflicted impairments, and LiSat-11 would indicate domains in which the patient is dissatisfied and therefore likely to be candidates for intervention in a rehabilitation program. Nevertheless, it is likely that some LiSat-11 domains overlap with individual MPI items, i.e. answer the same question, whereas for others they may capture true different aspects.

### Association of LiSat-11 domains to individual MPI scales

The first approach was to search for individual MPI scales which were clearly linked to high or low life satisfaction. Most correlations between individual MPI scales and LiSat-11 items were weak, indicating that many of the functional impairments as measured by MPI are not necessarily linked to strong impact on life satisfaction. However, we found three correlations that were more pronounced than others. Firstly, MPI life control correlated well to LiSat-11 psychological health. This correlation is in good accordance with the basic general ideas of improving sense of control as a mean to improve the perception of health. Secondly, MPI scale punishing responses from near relatives was negatively correlated to LiSat-11 partner relationship. This finding was not unexpected either but nevertheless indicates the importance of involving relatives in the rehabilitation strategies. Third, also not unexpected, the MPI scale affective distress was negatively correlated to LiSat-11 psychological health.

Interestingly, satisfaction with vocation, economy and activities of daily living was not correlated to any of the MPI scales. We do not know at this point whether they would have judged this domain differently without financial support from the National Insurance system.

Special interest was focused on the relation of reported pain severity to the different LiSat-11 domains. We used the median value from the MPI scale "pain severity" to dichotomize the patients into those who reported more intense pain, and less intense pain. The result showed that lower pain severity tended to be associated only with higher satisfaction with somatic health and psychological health. The patients in the present study were probably more affected by pain than patients with long term pain in general, as they had been referred to a multidisciplinary team rehabilitation. For this reason, the results in this study may not represent patients with long-term pain in general.

Like life satisfaction, it has repetitively been shown that HRQL is comparatively low among patients with long-term pain, and also that other factors than pain severity, such as catastrophizing[24,25] may predict quality of life even better than pain severity. Previous studies have also indicated that pain intensity is poorly correlated to physical impairment[26]. Together, this indicates that pain severity alone is not as strong a predictor of the level of life satisfaction/quality of life among patients with long-term pain as might be expected, and that other factors should be evaluated as well. This does not exclude the possibility that interventions to reduce pain severity might increase the level of life satisfaction in individual patients. Pain reduction has been associated with increased quality of life after treatment with for instance a coxiber[27], and fentanyl[28].

### Association of LiSat-11 domains and MPI-S subgroups AC, DYS, and ID

The second approach was to study life satisfaction in the three MPI-S subgroups: AC, DYS, and ID patients. These subgroups relate to different categories of patient behaviour and therefore may be more meaningful for comparison with life satisfaction in a clinical setting than individual MPI scales.

MPI and/or MPI-S has previously proved to be useful to describe impact of pain in patients with non-specified pain[10], temporomandibular joint disorder [29-31], patello-femoral syndromes[32], pain related to post-polio syndrome[33], low back pain[34] spinal cord injury[35], and whiplash associated disorder[12]. For whiplash associated disorder, patients belonging to the different MPI-S subgroups were found to differ with regard to self efficacy, disability and coping measure[12]. Furthermore, the MPI scale interactions were found to be a strong predictor for development of long-term pain after whiplash injury[36]. MPI subgroups show association to psychiatric co-morbidity in fibromyalgia patients; DYS patients have more anxiety, and ID more depression, whereas AC are comparatively well[37].

Here we used only the first two of the three MPI sections, as the third (activity) showed low internal validity. Multivariate analysis followed by univariate tests showed that differences existed between three similar subgroups in our material, and pair-wise comparisons confirmed the validity among the patients in the present study. Corresponding MPI subgroups have also been described by Graded Chronic Pain scale GCP, at least for patients with tempero-mandibular joint pain[38].

In the present study AC patients reported higher life satisfaction than the DYS and ID patients in all LiSat-11 domains, except family life and partner relationship for which such difference between AC and DYS could not be established. This finding seems to be in correspondance with the findings of Bergström et al[11] who showed that the AC patients had fewer absences from work and utilised health care less than the DYS patients. However, our findings also indicate that even for AC patients who otherwise seem to be better off than the DYS and ID patients, efforts to improve family and partner relationships may be important and may deserve attention in a rehabilitation program. In fact, when we added all ten LiSat-11 domains and correlated the sum to satisfaction with life as a whole, we found that among the patients with chronic pain, satisfaction with family life and with sexual life showed the strongest correlations.

### Clinical implications

It has previously been suggested that DYS patients benefit most from a combination of physical therapy and cognitive behaviour therapy[11] whereas ID patients need their interpersonal and/or marital problems to be addressed. Our results support these ideas by indicating that the problems linked to those subgroups are associated also by trends of low satisfaction. The ID patients in particular may need to involve their relatives in the rehabilitation process.

The results of this study may indicate less direct needs of intervention for AC patients. However, it is also possible that they are in need of interventions to help them to remain in that subgroup "have more to lose". Previous studies have shown that MPI subgroups may change with time. AC patients becomes fewer and the ID patients increase[39], perhaps indicating transition form AC to ID for some patients.

The predictive value of MPI subgroups vary in reports of outcome after treatment and rehabilitation for long-term pain. MPI subgroups did not predict differential outcome after a fibromyalgia program[40], a medicine program for patients with migraine[41], an interdisciplinary pain program for patient with heterogeneous diagnoses[42], or a vocational rehab program[11]. Outcome studies of patients with tempero-mandibular joint disorder[43] and fibromyalgia[44] showed that DYS patients tended to benefit more than AC and ID from a standardized treatment program. This does not exclude that the outcome would have been better if the programs were designed to meet the requirements of each MPI subgroup. Whether such specialised programs for MPI subgroups would be an efficient approach remains to be shown. There do not seem to be any studies on the predictive value of LiSat-11 in outcome studies after treatment or rehabilitation of patients with long-term pain.

## Conclusion

The strongest positive correlation were found for the LiSat-11 domains/MPI scales: psychological health/life control and contacts/social activities, and the strongest negative correlation for: psychological health/affective distress, partner relationship/punishing responses, somatic health/interference and leisure/interference. The latter may indicate domains that need to particular attention in rehabilitation programs. Furthermore, none or only little correlation was found between MPI scale pain severity and most LiSat-11 domains and satisfaction with life as a whole. This finding raises the question of the value of partial pain relief alone for these patients. Patients belonging to the MPI-S subgroup "adaptive copers" had higher satisfaction than "interpersonally distressed" and "dysfunctional" on most LiSat-11 domains. This may indicate that individual rehabilitation programs designed to meet the need each of these MPI-S subgroups are required.

## Competing interests

The authors declare that they have no competing interests.

## Authors' contributions

AJS contributed to the final designing of the project, collected patient data and drafted the manuscript. HK contributed to the design of the project, performed statistical analysis, and contributed to the manuscript. KP was involved in the initiation of the project and contributed to the manuscript. CM contributed to the final designing of the project and to the drafting of the manuscript. All authors commented on the drafts of the manuscript and read and approved of the final version.
